# Supplementary material for: A Retrospective Analysis of 10 Years of Liver Surveillance Undertaken in Uveal Melanoma Patients Treated at the Supraregional “Liverpool Ocular Oncology Centre”, UK
Source: Cancers (Basel). 2022 Apr 27;14(9):2187. doi: 10.3390/cancers14092187 (PMC9102800; doi:10.3390/cancers14092187)
Supplement: Supplementary file 1 [file cancers-14-02187-s001.zip › cancers-1665374-supplementary.pdf]

cancers

MDPI

Supplementary Material: A Retrospective Analysis of 10 Years of Liver Surveillance Undertaken in Uveal Melanoma Patients Treated at the Supraregional "Liverpool Ocular Oncology Centre", UK

Alda Rola, Helen Kalirai, Azzam F. G. Taktak, Antonio Eleuteri, Yamini Krishna, Rumana Hussain, Heinrich Heimann and Sarah E. Coupland

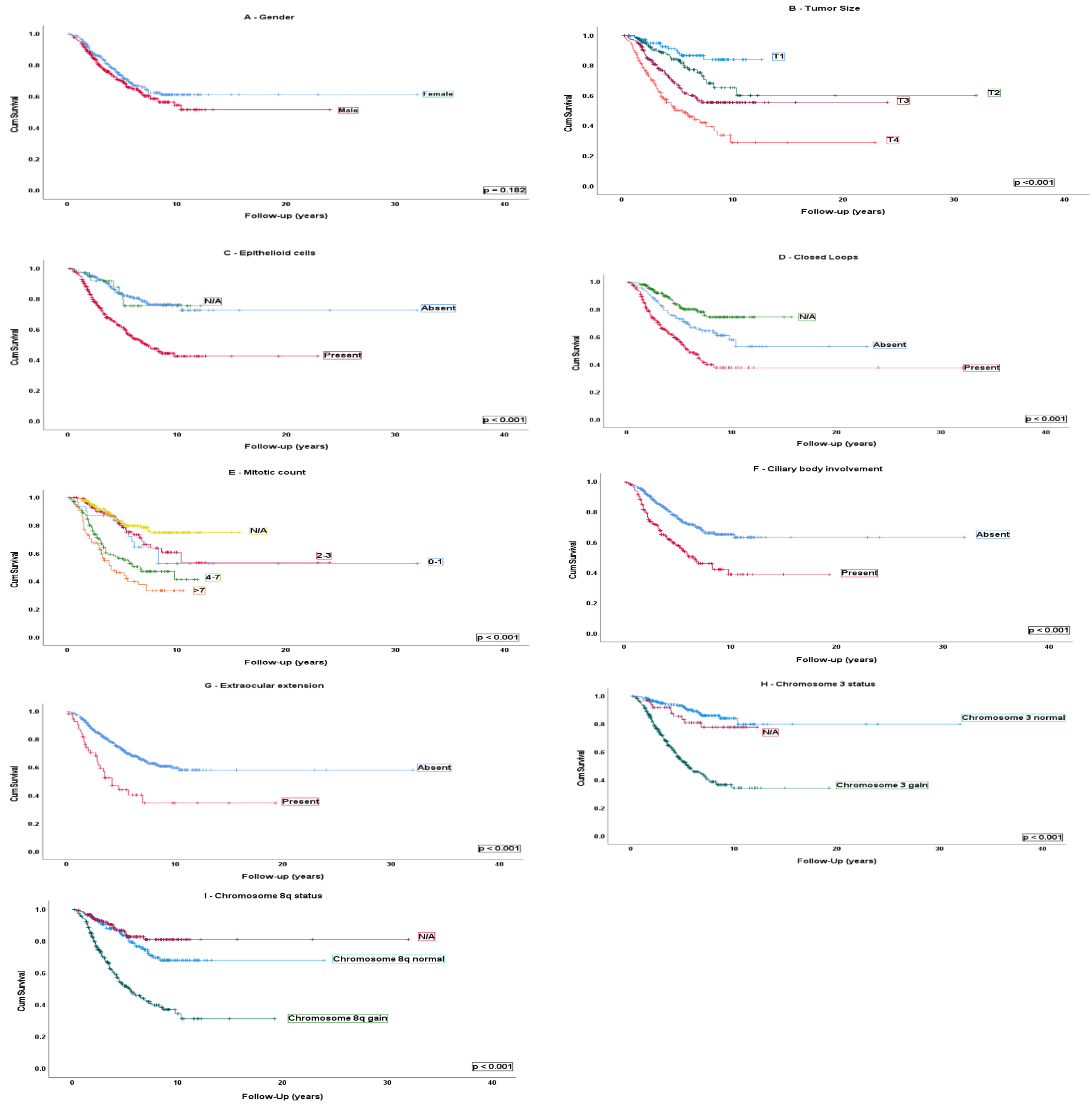

Figure S1: Kaplan-Meier plots (A-I). Overall survival for all patients stratified according to all predictor factors related to LUMPO3. All these predictor factors were associated to poor survival and metastatic mortality. A – Male gender, B – Increasing tumor size, C – presence of epithelioid cells, D – presence of closed loops, E – High mitotic count, F – Ciliary body involvement, G – Extraocular extension, H – Chromosome 3 alterations, I – Chromosome 8q alterations.

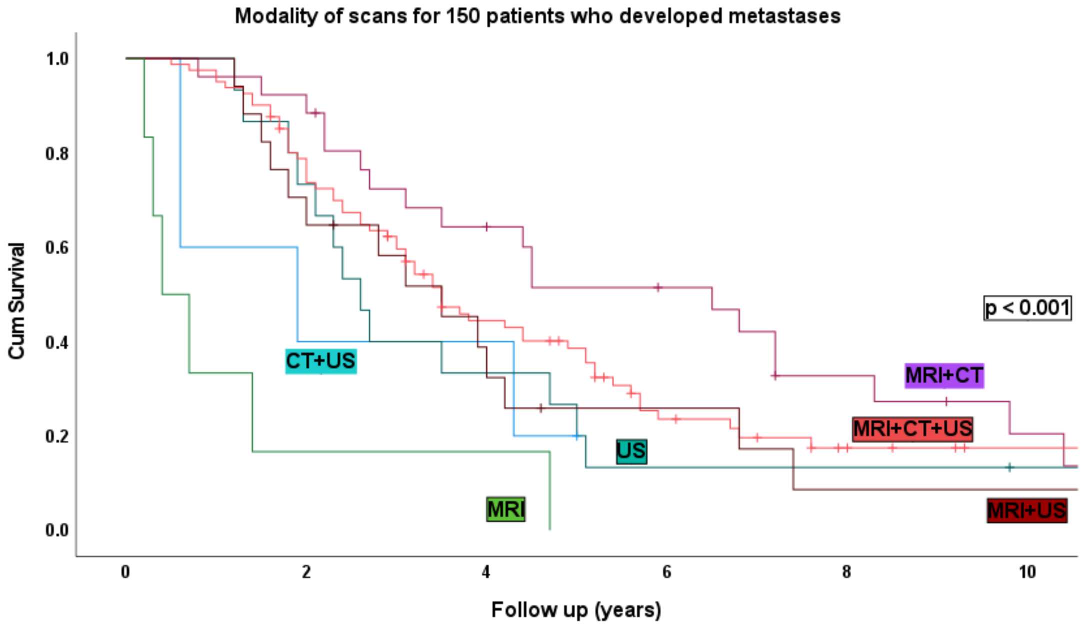

Modality of scans	Number of patients	(%)	Number of Scans	(%)	Number of Events	(%)	Censored	(%)	Median survival (years)	95% confidence interval	
										Lower	Upper
**MRI**	15	(10%)	74	(5%)	13	(87%)	2	(13.3%)	2.600	2.095	3.105
**US**	6	(4%)	6	(0.4%)	6	(100%)	0	(0%)	0.400	0.000	0.880
**MRI + CT**	26	(17%)	297	(19%)	19	(73%)	7	(26.9%)	6.500	3.817	9.183
**MRI + US**	17	(11%)	129	(8%)	14	(82%)	3	(17.6%)	3.500	2.123	4.877
**CT + US**	5	(3%)	27	(1.7%)	4	(80%)	1	(20.0%)	1.900	0.000	4.691
**MRI + CT + US**	81	(50%)	1067	(67%)	59	(73%)	22	(27.2%)	3.500	2.875	4.125
**Overall**	150		1600		115	(77%)	35	(23.3%)	3.500	2.875	4.125

Figure S2. Kaplan-Meier survival curve and table where 150 UM patients with metastases. detected through liver surveillance were stratified according to the modality of scan performed. MRI: 15 patients underwent 74 scans; US: 6 patients underwent 6 scans; MRI+CT: 26 patients underwent 297 scans; MRI+US: 17 patients underwent 129 scans; CT+US: 5 patients underwent 27 scans; MRI+CT+US: 81patients underwent 1067 scans. Number of events indicates the number of deaths from metastatic disease.

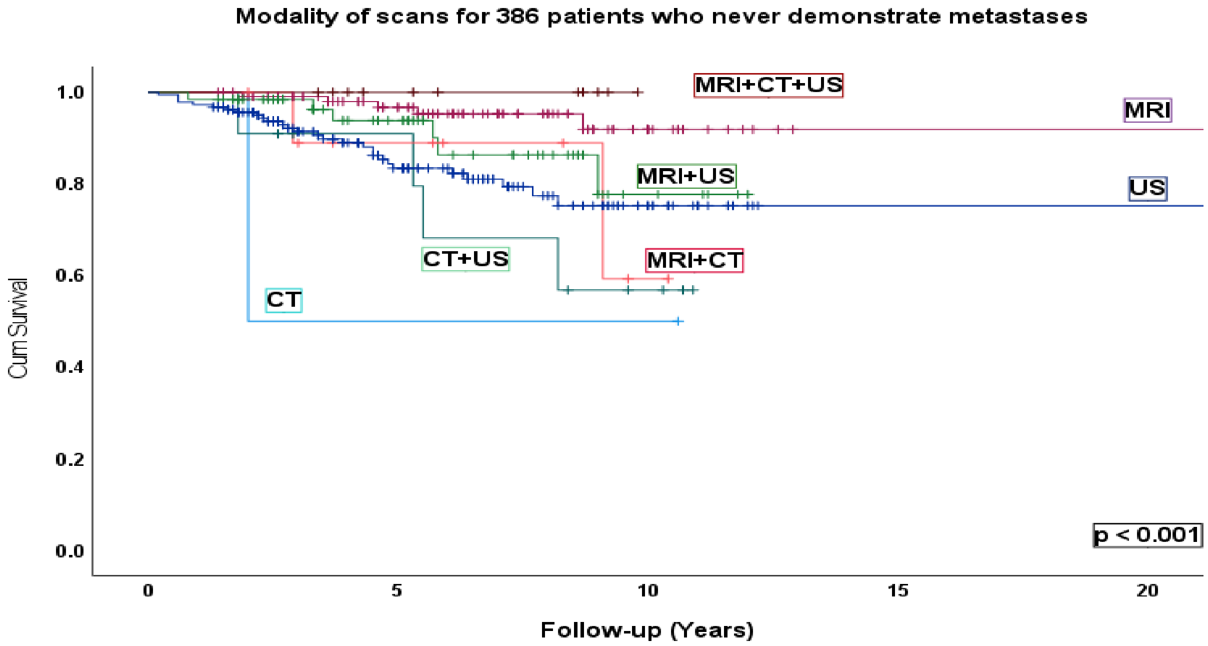

Modality of scans	Number of patients	(%)	Number of Scans	(%)	Number of Events	(%)	Censored	(%)	Median survival (years)	95% confidence interval	
										Lower	Upper
**MRI**	107	(28%)	871	(41%)	5	(87%)	102	(95.3%)	-	-	-
**CT**	2	(0.5%)	6	(0.3%)	1	(2.1%)	1	(50.0%)	-	-	-
**US**	183	(47%)	456	(22%)	28	(100%)	155	(84.7%)	-	-	-
**MRI + CT**	10	(3%)	103	(5%)	2	(73%)	8	(80.0%)	-	-	-
**MRI + US**	62	(16%)	488	(23%)	6	(82%)	56	(90.3%)	-	-	-
**CT + US**	11	(3%)	53	(3%)	4	(80%)	7	(63.6%)	-	-	-
**MRI + CT + US**	11	(3%)	137	(6%)	0	(73%)	11	(100%)	-	-	-
**Overall**	386		2114		46	(77%)	340	(80.1%)	-	-	-

Figure S3. "Kaplan-Meier survival curve and table where 386 UM patients who never demonstrated metastases were stratified according to the modality of scan performed". MRI: 107 patients underwent 871 scans; CT: 2 patients underwent 6 scans; US: 183 patients underwent 456scans; MRI+CT: 10 patients underwent 103 scans; MRI+US: 62 patients underwent 488 scans; CT+US: 11 patients underwent 53 scans; MRI+CT+US: 11 patients underwent 137 scans. No survival statistics because all cases are censored. Number of events indicates the number of deaths.

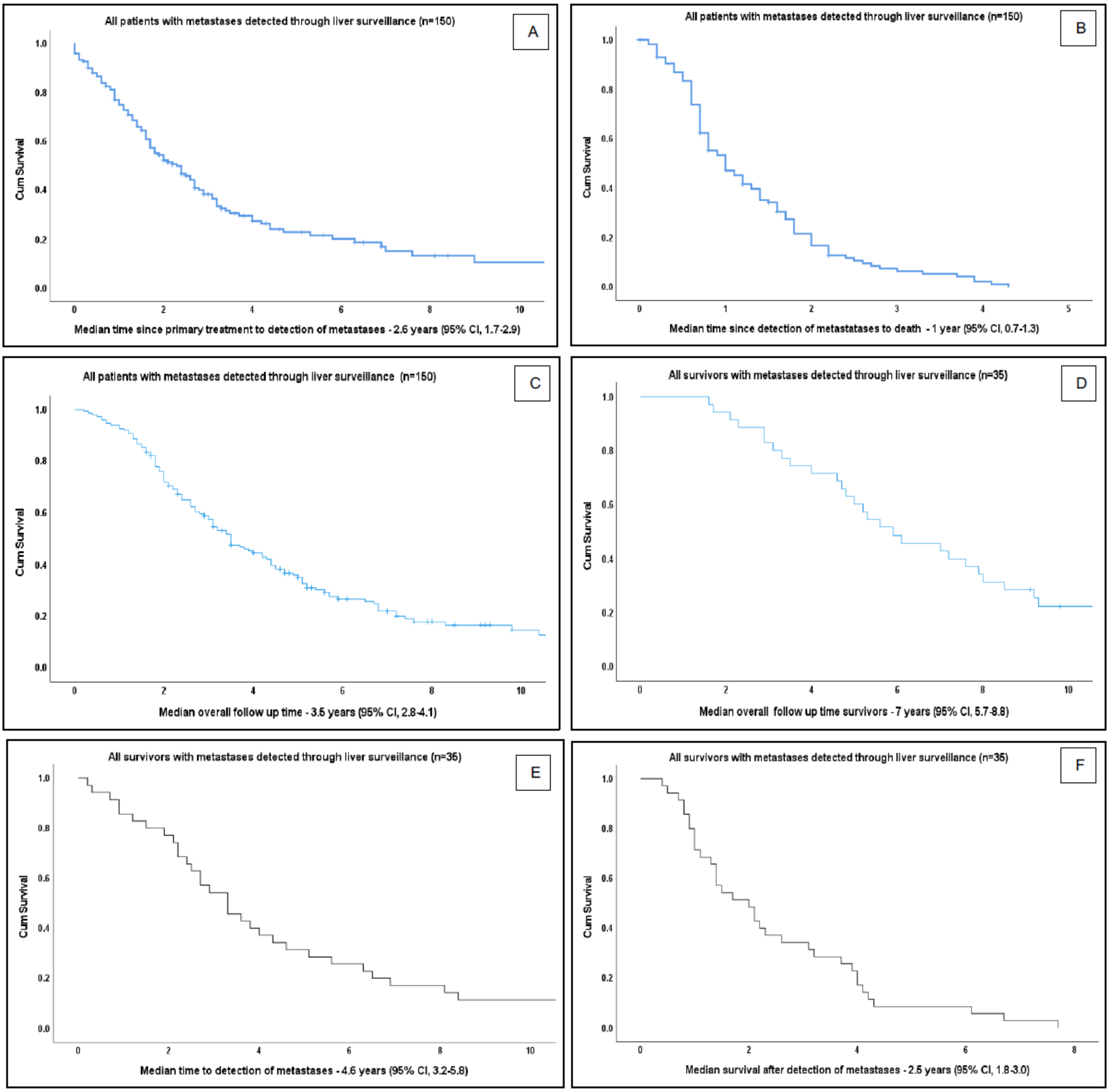

Figure S4. Kaplan Meier estimates for all 150 metastatic UM patients from primary treatment to detection of metastases, and from metastatic disease to death. A) Median time to detection of metastases. B) Median time from detection of metastases to death. C) Median follow-up time for all patients. D) Median overall follow-up time for survivors. E) Median time to detection of metastases for survivors. F) Median follow up time for survivors after detection of metastases.

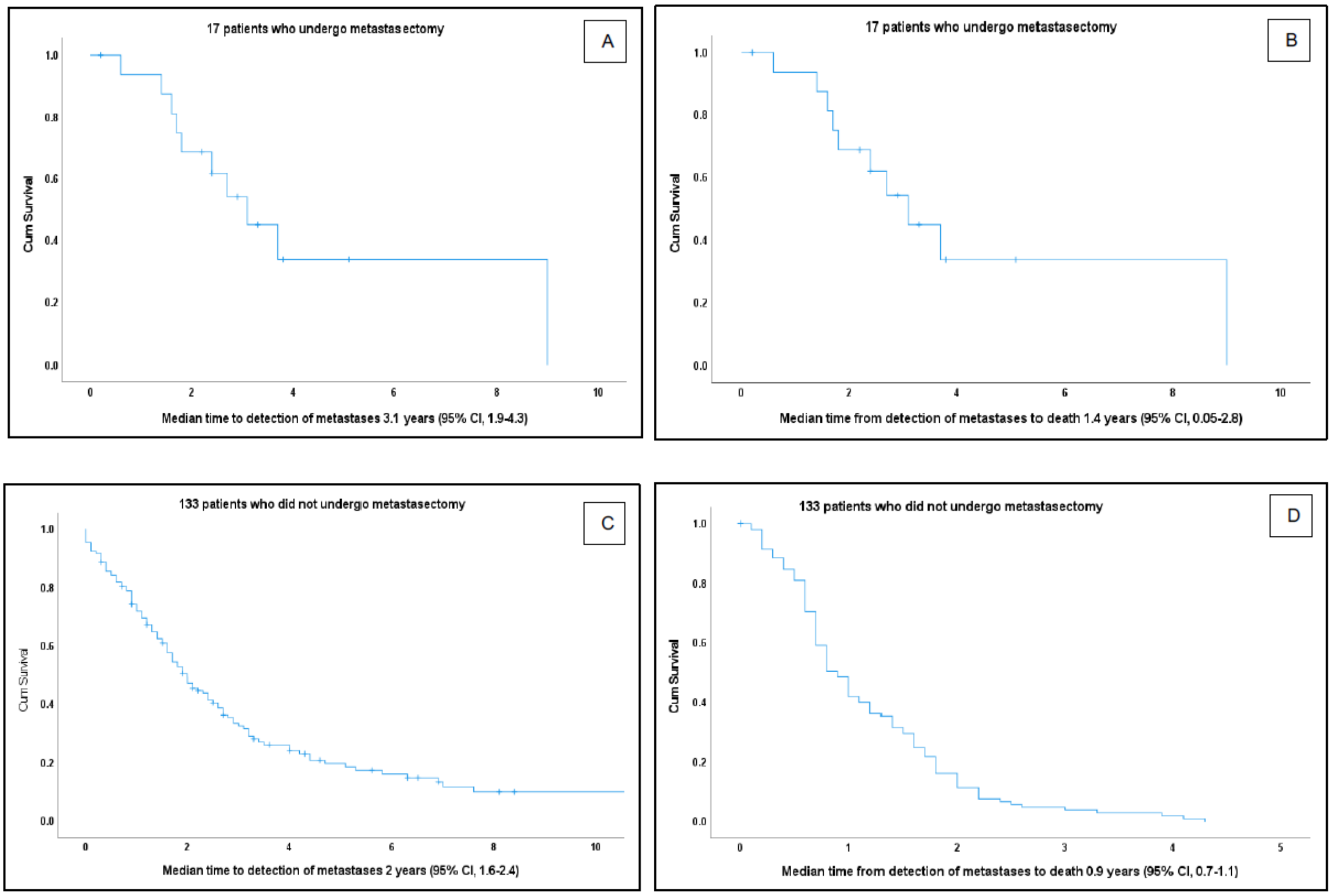

Figure S5. Kaplan-Meier estimates for all 150 metastatic UM patients stratified according to type of treatment post diagnosis of metastases. Metastasectomy was performed in 17 patients, while in the remaining 133 patients no metastasectomy event was recorded. Patients undergoing metastasectomy: A) Median time to detection of metastases, B) Median time from detection of metastases to death. Patients who did not undergo metastasectomy: C) Median time to detection of metastases, D) median time from detection of metastases to death.

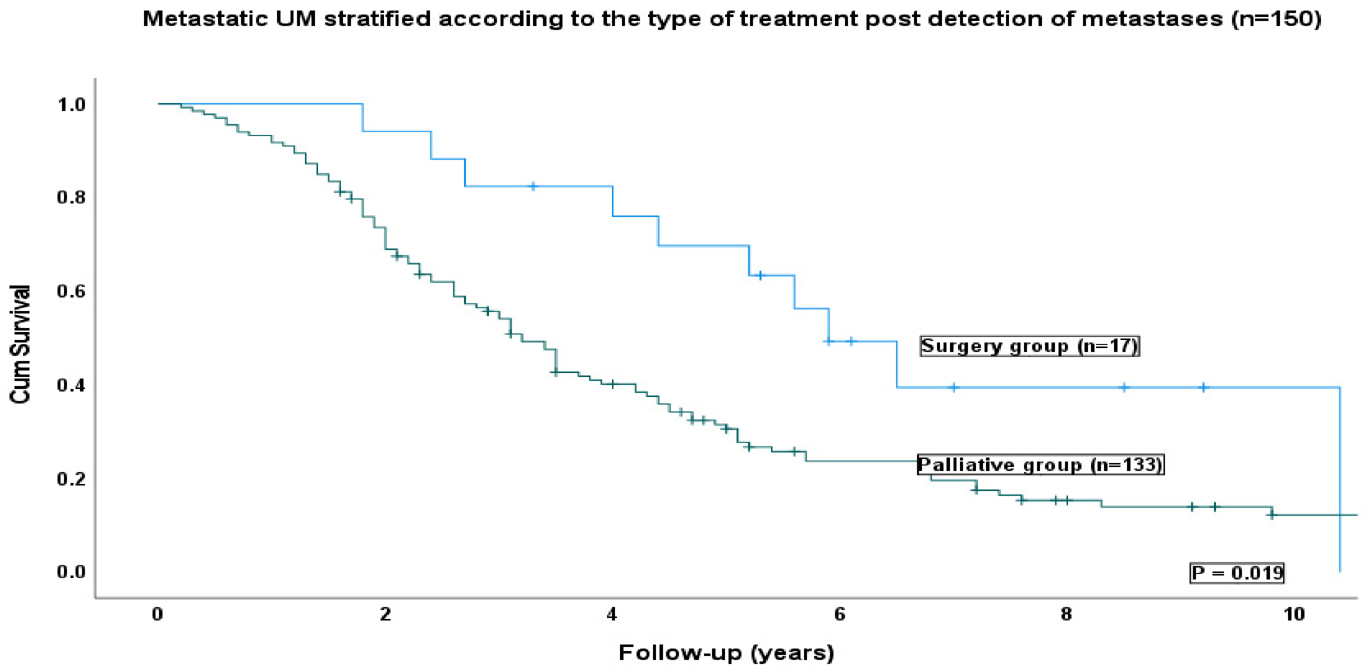

Groups	Number of patients (%)		Number of events %		Censored %		Sig.	Median survival (years)	95% confidence interval	
									Lower	Upper
Surgery	17	11%	10	59%	7	41.2%	P = 0.019	5.900	4.564	7.236
Palliative	133	89%	105	79%	28	21.1%	P = 0.019	3.200	2.803	3.597
Overall	150	100%	115	77%	35	23.3%		3.500	2.875	4.125

Figure S6. Kaplan-Meier survival curve and table for all metastatic UM stratified according to the type of treatment post detection of metastases. Patients were classified as: Surgery group (n = 17), and palliative group (n = 133), (p = 0.019).

Table S1. Summary of studies reporting outcome of UM metastatic disease.

Parameters	Studies				
	**Cunha Rola et al.**	**Rietschel et al.**	**Rivoire et al.**	**Lane et al.**	**Lorigan et al.**
**Name of the Study**	A retrospective analysis of 10 years of liver surveillance undertaken in UM patients treated at the supraregional LOOC	Variates of survival in metastatic UM	Treatment of liver metastases from UM	Survival rates in patients after treatment for metastases from UM	The prevalence and location of metastases from ocular melanoma: Imaging study 110 patients
**Number of UM patients**	615 UM patients	119 (stage IV UM)	602	3063	181
**Time of the study**	11 years	10 years	14 years	27 years	12 years
**Metastatic disease**	Total 229 (37%) UM metastases:<br>150 (66%) imaging<br>79 (34%) post-mortem	119 – UM metastases<br>77% - liver<br>43% - other sites	10.5% UM metastases	661 (21.6%)<br>12 (1.8%) excluded<br>87 (13%) no data<br>562 (85%) Metastases	110 (60.7%) MUM<br>101 (92%) liver + other sites<br>60 (55%) only liver
**Metastases at time of diagnosis UM**	7 (2.4%)	N/A	N/A	12 (1.8%) metastases within 6 months	3 (2.7%)
**Parameters of the study**	Diagnosed by imaging 65.5 %<br>Diagnosed at autopsy 34.5 %	Diagnosed symptoms 40%<br>Diagnosed imaging/others 60%	44% Liver surgery<br>56% Systemic chemotherapy	53% received treatment<br>47% untreated	Clinical and radiology reports

**Time to metastases**	2.6 years (0.1-17.8)	4.4 years (0-29.9)	2.4 years	3.45 years	4.3 years (0.2-36)
**Time to death**	12 months (1-52 months)<br>< 2 years – 85%<br>2-3 years - 10%<br>>3 years – 5%	9.5 months - Systemic treatment<br>32.4 months -Surgery/intrahepatic	13 months (2.5-110)<br>1 year – 59%<br>2 years – 29%<br>4 years – 13%	12 months– 21%<br>1.5 years – 11%<br>3 years – 4%	10 months (1-38) – 96%
**Median survival after metastases**	12 months<br>(95% CI, 0.7-1.3)	12.5 months<br>(95% CI, 9.5-15.7 months)	15 months<br>(3-110 months)	3.9 months – received treatment<br>1.7 months – no treatment	N/A
**Overall survival patients with metastases**	3.5 years (95% CI, 2.8-4.1)	N/A	3.9 years (0.3-15.8)	8.9 years<br>(0.1-29.6 years)	N/A
**Survivors**	(23.3%)	(22%)	(15.9%)	(30.2%) treated alive<br>(5.6%) untreated alive<br>After 1 year of treatment	(4.5%)<br>(2.7%) – 22 months<br>(1.8%) – 74 months
**Median Follow-up survivors**	30 months<br>(4-92 months)	17 months<br>(0-96 months)	29 months<br>(16-110 months)	N/A	N/A
